# Can surgical treatment be justified for neuroendocrine carcinoma of the gallbladder?

**DOI:** 10.1097/MD.0000000000014886

**Published:** 2019-03-15

**Authors:** Yung Hun You, Dong Wook Choi, Jin Seok Heo, In Woong Han, Seong Ho Choi, Kee-Taek Jang, Sunjong Han

**Affiliations:** a Department of Surgery, Konkuk University Choongju Hospital, Konkuk University School of Medicine, Chungju-si, Chungcheongbuk-do; b Department of Surgery, Samsung Medical Center, Sungkyunkwan University School of Medicine, Gangnam-gu, Seoul; c Department of Surgery, Samsung Changwon Hospital, Sungkyunkwan University School of Medicine, Masanhoewon-gu, Changwon-si, Gyeongsangnam-do; d Department of Pathology and Translational Genomics, Samsung Medical Center, Sungkyunkwan University School of Medicine, Gangnam-gu, Seoul; e Department of Surgery, Seoul National University College of Medicine, Surgery, Seoul National University Bundang Hospital, Bundang-gu, Seongnam-si, Gyeonggi-do, South Korea.

**Keywords:** clinical features, gallbladder neuroendocrine carcinoma, surgical treatment

## Abstract

Clinical features and treatment of GB neuroendocrine carcinoma (GB-NEC) are not well understood. This study aimed to analyze clinical outcomes of GB-NEC and verify the oncologic benefit of surgical treatment.

From October 1994 to December 2014, the medical records of 31 patients with GB-NEC at a single center were retrospectively reviewed. There were 18 inoperable cases due to distant metastasis, including 7 of best supportive care (Tx.1) and 11 of non-operative palliative treatment (Tx.2). 4 patients received non-curative, palliative resection (Tx.3). Only 9 patients were able to undergo curative-intent resection (Tx.4).

Among the 31 patients with GB-NEC, preoperative mean value of carbohydrate antigen 19-9 (CA 19-9) was 74.8 ± 156.1 U/mL and the median overall survival time was 10 months (range 7.0-12.0 months). Of these, 21 (67.7%) patients received systemic treatment. Among 9 patients who underwent curative-intent resection (Tx.4), 9 patients had poorly differentiated cancer cells and 7 patients received radical cholecystectomy. 6 patients had adjuvant treatment including concurrent chemoradiation therapy (CCRT) or chemotherapy alone. The recurrence rate was 88.9%. The median overall survival between 4 groups was as follows: 4.0 (3.0–18.0) months in Tx.1 (n = 7) versus 9.0 (3.0–21.0) months in Tx.2 (n = 11) versus 11.0 (3.0–15.0) months in Tx.3 (n = 4) versus 23.0 (8.0–34.0) months in Tx.4 (n = 9), respectively. Significant differences in median overall survival time existed between Tx.2 and Tx.4; 9 (3.0–21.0) months versus 23.0 (8.0–34.0) months (*P* = .017).

Most GB-NECs show poor biologic behavior. Nonetheless, curative-intent resection could possibly promote longer survival than other treatment modalities for GB-NEC. Efforts to undergo curative resection through early detection and development of adjuvant treatment are needed.

## 1 Introduction

Neuroendocrine tumors (NETs) are malignant disease and develop usually along gastroenteropancreatic sites.^[1,2]^ Biliary NETs account for less than 1% of all NETs.^[1,3,4]^ Among these, gallbladder neuroendocrine tumors (GB-NETs) are particularly rare.^[5]^ The Surveillance, Epidemiology, and End Results (SEER) registry reported that merely 278 cases of GB-NETs occurred between 1973 and 2005, accounting for 0.5% of all NET cases.^[6,7]^ Based on World Health Organization (WHO) 2010 classification,^[8]^ NETs are composed of neuroendocrine tumor grade 1 (NET G1), neuroendocrine tumor grade 2 (NET G2), neuroendocrine carcinoma grade 3 (NEC G3); large cell or small cell type and mixed adeno-neuroendocrine carcinoma (MANEC). Specifically, 66% of NECs arises in the gastrointestinal (GI) tract, and NECs in the extrahepatic duct and gallbladder (GB) represent only 0.2% to 2% and 0.2% of all GI tract NECs.^[6,9]^

A very low incidence of GB carcinoma (GB-NECs) made it difficult to analyze clinicopathologic features of disease and establish a treatment strategy. There were only a few case reports and investigations analyzing clinical outcomes of GB-NEC. As a result, the purpose of this study is to uncover clinicopathologic features and survival outcome of GB-NEC Finally, this study attempts to illuminate new details regarding this unfamiliar disease and validate effect of provide proper treatment modality.

## 2 Methods

### 2.1 Methods and patients selection

From 1994 to 2014, 31 patients were diagnosed with GB-NEC at a single center. One pathologist (Jang KT) confirmed all reports and findings. The data from these patients were prospectively collected in electronic medical record form and retrospectively reviewed. The demographics and clinical features of 31 GB-NEC cases were summarized in Table 1. This study was approved by Institutional Review Board of Samsung Medical Center, Seoul, Republic of Korea (approval number: 2017-12-046)

**Table 1:**
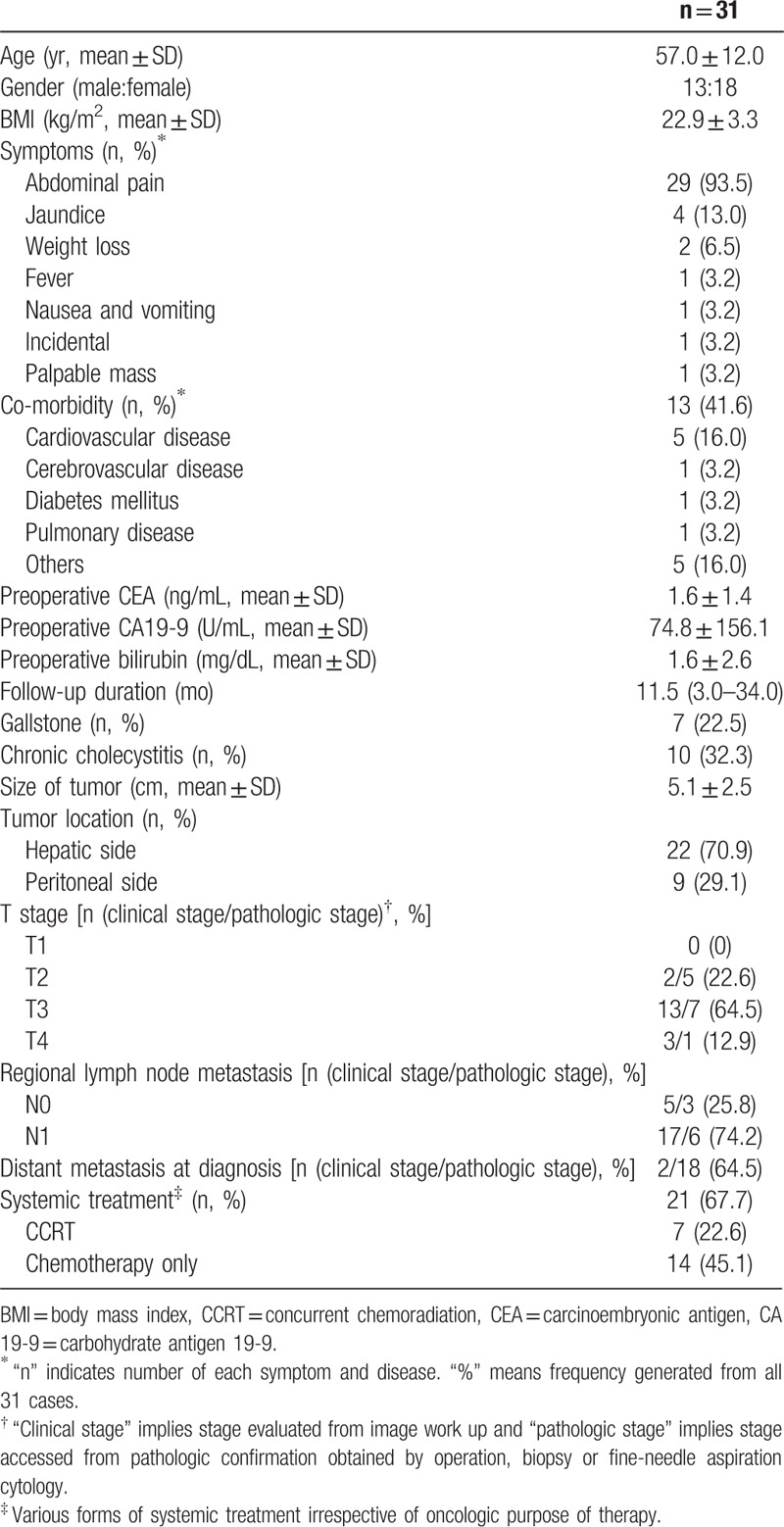
Demographics and clinical features of GB-NEC (31 cases).

### 2.2 Definition of treatment

**Figure 1:**
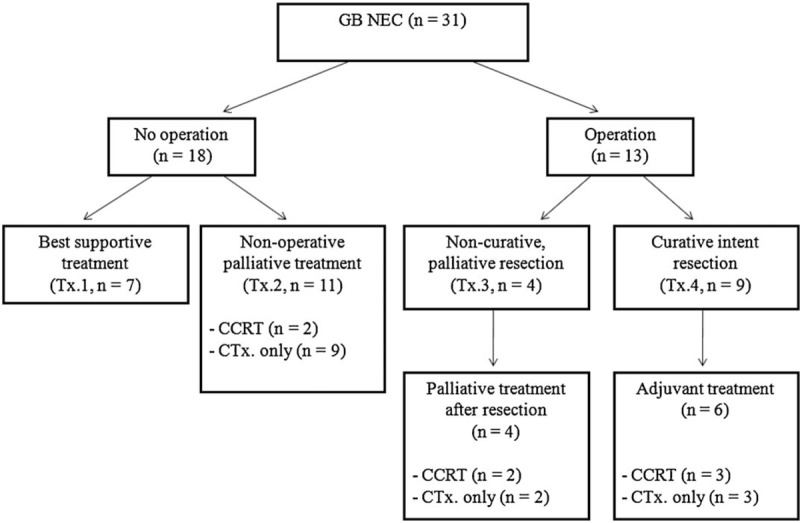
Treatment in patients diagnosed with GB-NEC. CCRT = concurrent chemoradiation treatment.

Figure 1 illustrates treatment in 31 patients who were diagnosed with GB-NEC. Of these patients, 18 patients did not undergo surgery because distant metastasis was confirmed by pathologic examination. The tissues were obtained by biopsy and/or fine-needle aspiration cytology. Among these, 7 patients did not want any further palliative treatment and only received the best supportive care (Tx.1), consisting of emotional support and medical treatments for symptom control such as analgesics, antibiotics, antiemetics, or antidiarrheals without surgery, chemotherapy or concurrent chemoradiation therapy (CCRT). In contrast, palliative chemotherapy or CCRT was carried out in 11 patients with consent from patients and guardians. This was defined as non-operative palliative treatment (Tx.2). The remaining 13 patients were able to undergo surgery. Among these, 4 patients underwent non-curative, palliative resection (Tx.3), of whom 2 received palliative cholecystectomy for tissue confirmation or resolving the problem of perforation at the GB, despite detection of distant metastasis by preoperative computed tomographic scan. The other 2 cases did not exhibit distant metastasis on a preoperative image examination. However, 1 patient underwent palliative cholecystectomy due to difficulties of approach to a hepatoduodenal ligament despite positive cancer presence in the cystic duct upon frozen examination. Another patient was 78 years old and had ischemic heart disease. We underwent palliative cholecystectomy considering high risk of perioperative morbidity and life expectancy. Finally, only 9 patients were able to receive curative intent resection (Tx.4).

### 2.3 Preoperative work up and operation methods

We performed abdominal computed tomographic examination for evaluation of respectability and formulating a surgical strategy. Assessment of tumor depth, invasion to adjacent organ and evidence of distant metastasis was done through image work up preoperatively. If no distant metastatic lesion was shown and tumor resectability was confirmed, curative intent operations were performed. The extent of resection was determined by the surgeon's comprehensive evaluation based on intraoperative gross findings of cancer and state of the patient. First, if the depth of invasion appeared to be more than a T2 lesion, a radical cholecystectomy was performed, including removal of the GB, regional lymph node, and liver close to the GB bed. In such cases, the frozen examination in reference to depth of invasion was not performed. However, if there is uncertainty in the depth of invasion exceeding a T2 lesion, frozen examination was performed for further analysis. In case of more than a T2 lesion confirmed on frozen examination, radical cholecystectomy was done. The extent of liver and GB bed resection was more than 2 cm in depth of GB bed or bisegmentectomy of segment 4b and 5 respectively. If the tumor invades the hilar area, proximal common bile duct or adjacent major vessel like hepatic artery or portal vein, hemihepatectomy or extended hemihepatectomy was considered.

### 2.4 Histopathologic examination and cancer staging

Immunohistochemical examination of synaptophysin (Syn), chromogranin A (CgA), neuronspecific enolase (NSE), Ki-67, cluster of differentiation 56 (CD56), cluster of differentiation 99 (CD99), p53, transmembrane glycoprotein Mucin 1 (MUC1), and transmembrane glycoprotein Mucin 6 (MUC6) was performed. The diagnosis of GB-NEC was based on the WHO classification published in 2010.^[8]^ Among them, diagnostic criteria of small cell NEC of the GB (GB-SCNEC) was as follows:

(1) NET with >10 mitoses/2 mm^2^ and small cell cytological features.^[7,10,11]^
(2) positive findings of more than 1 protein, including CgA and Syn, or CD 56, known as neuroendocrine markers in immunochemistry.^[7,10]^

The diagnosis of large cell NEC of the GB (GB-LCNEC) is performed by following criteria:

(1) NET with >10 mitoses/2 mm^2^ and cytologic features of a large cell carcinoma^[12]^
(2) strong cytoplasmic staining for neuroendocrine markers (CgA and Syn) in immunochemistry.^[10]^

The definition of MANEC is a carcinoma that is a mixture of 2 morphologically recognizable phenotypes, including both gland forming epithelial and neuroendocrine cells, and satisfies a condition that at least 30% of either component exist.^[8]^ The 7th edition of TNM staging for GB cancer issued by the American Joint Committee on Cancer (AJCC) was utilized for cancer staging.

### 2.5 Follow-up

Follow-up examinations were performed at 3-month intervals for the first 12 months after operation, and then decreased to 6 months intervals. In the case of no recurrence for 18 months after resection, follow-ups were maintained to 6-months. Follow-up studies were composed of image work ups, including computed tomography and laboratory findings such as tumor markers.

### 2.6 Definition of recurrence

Recurrence of disease was detected by radiologic or histologic findings during follow-up periods. The locoregional recurrence was defined as follows:

(1) local ill-defined mass or soft tissue on hepatic resection margin or bilioenteric anastomosis site
(2) increase in size of lymph node along porta hepatis or retroperitoneal lymph nodes.

By contrast, if the suspicious radiologic findings on intrahepatic, peritoneum, lymph node beyond locoregional area or extra-abdominal sites were detected or pathologic confirmation was done, it was classified as a distant recurrence.

### 2.7 Statistical analysis

Concerning cross-tabulate nominal data, Chi-square tests were implemented. Parametric continuous variables and nonparametric continuous variables were analyzed using Student *t* tests and the Mann–Whitney test, respectively. The analyses of disease-free and overall survival rates were performed using the Kaplan–Meier method and survival curves were compared using the log-rank test. Statistical significance was set at a value of *P* <.05. Data were analyzed using SPSS statistics version 23.0 (IBM corporation).

## 3 Results

### 3.1 The demographics and clinical features of GB NEC (31 cases)

The demographics and clinical features of 31 GB-NEC cases were summarized in Table 1. The mean age was 57.0 ± 12.0 years and 18 patients were female. The most common presenting symptom was abdominal pain, exhibited by 29 (93.5%) out of 31 patients. Preoperative mean value of carbohydrate antigen 19-9 (CA 19-9) was 74.8 ± 156.1 higher than the normal range (0-37 U/mL). Gallstone and chronic cholecystitis were found in 7 (22.5%) and 10 (32.3%) patients, respectively. The median follow-up duration was 11.5 months (range 3.0–34.0 months). The median overall survival time was 10 months (range 7.0–12.0 months). 21 (67.7%) patients received systemic treatment.

### 3.2 Clinicopathologic details of GB-NEC (Tx.4)

Table 2  summarizes characteristics and clinical outcomes of 9 patients with curative intent resection (Tx.4). The mean age was 56.7 ± 9.4 years and 7 patients were female. The most common presenting symptom was abdominal pain, exhibited by 6 out of 9 patients. Preoperative mean value of carcinoembryonic antigen (CEA) was within the normal range (0–3 ng/mL), while the mean value of CA 19-9 was above average (0–37 U/mL). Gallstone and chronic cholecystitis were found in 2 and 3 patients, respectively. The median follow-up duration was 18.6 months (range 8.0–34.0 months).

**Table 2:**
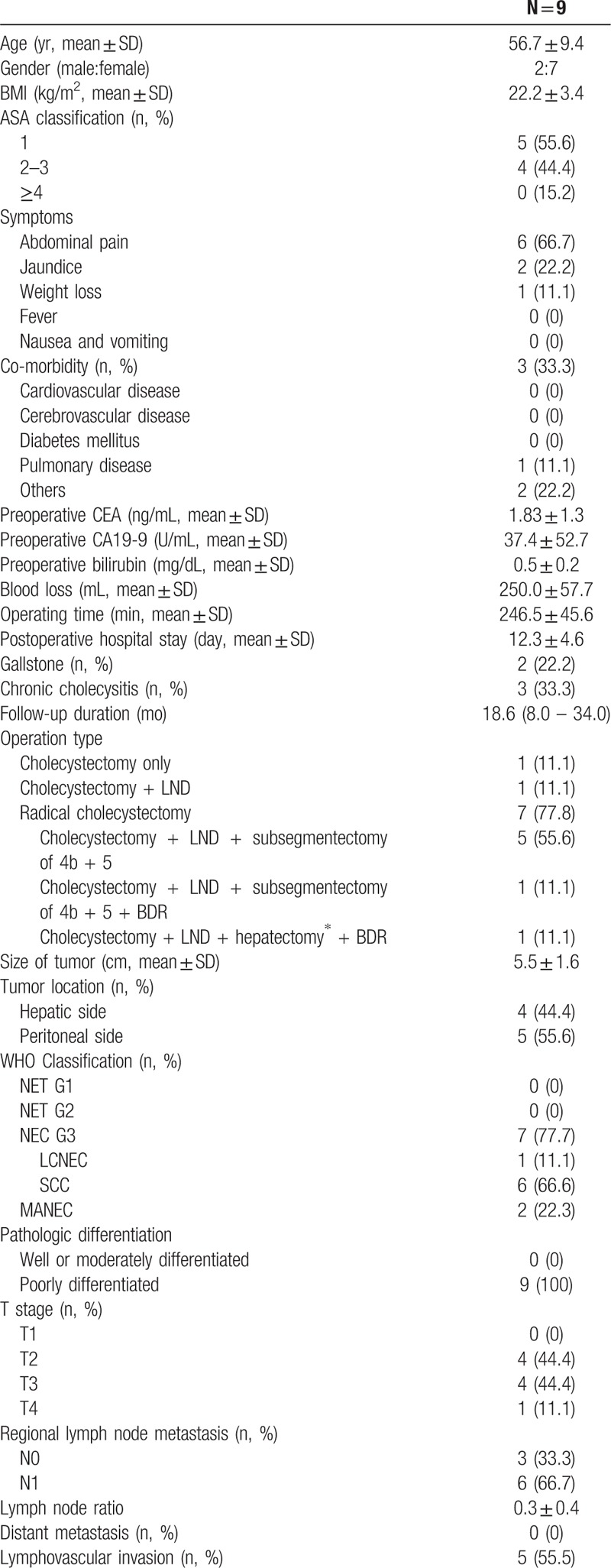
Clinicopathologic data of patients with curative intent resection (Tx.4).

**Table 2 (Continued):**
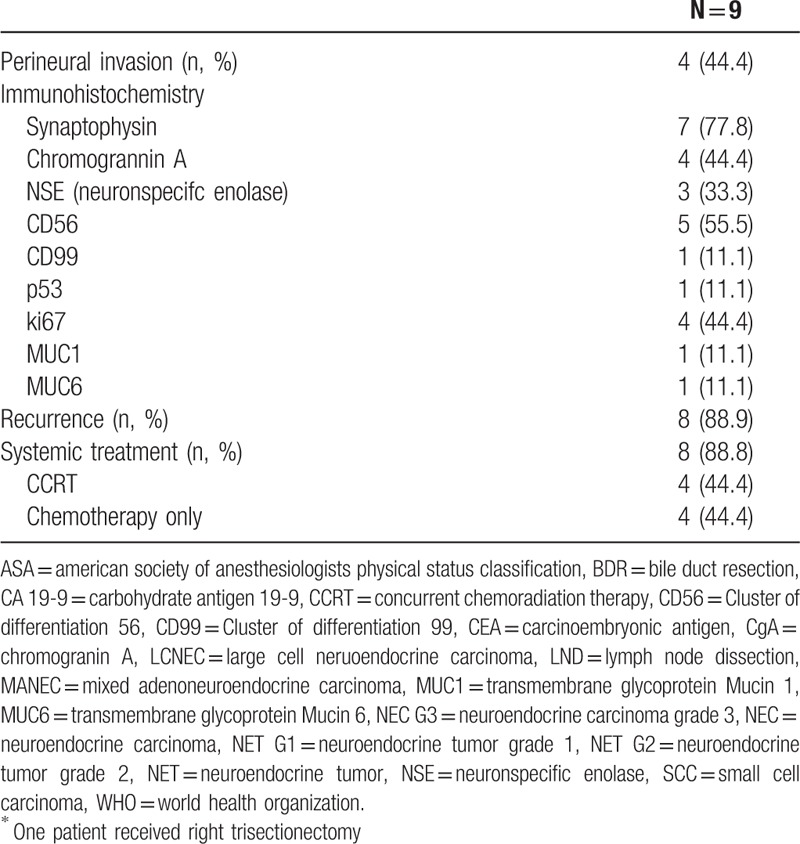
Clinicopathologic data of patients with curative intent resection (Tx.4).

Radical cholecystectomy was performed in 7 of the 9 patients. Among the 7 patients with extended cholecystectomy, 6 received segmentectomy of 4b and 5 and 1 patient underwent right trisectionectomy due to tumor invasion into the right lobe and perihilar area. Lymph node dissection including hepaticoduodenal ligament without hepatic resection was performed in 1 patient because a nonviable tumor was shown in the frozen biopsy of the GB neck. One patient underwent simple cholecystectomy under suspicion of cholecystitis and was diagnosed with GB-NEC incidentally as in final pathology.

There were 9 (100%) poorly differentiated tumors in all 9 cases. Of these cases, 6 showed GB-SCNEC and MANEC were found in 2 cases. The immunohistochemical expression of marker proteins was observed; 7 cases of Syn, 5 cases of CD56, 4 cases of chromogrannin A, 4 cases of Ki67 and 3 cases of NSE, respectively. Except for 3 cases, 6 patients had adjuvant treatment including CCRT or chemotherapy alone; 3 cases of CCRT and 3 cases of chemotherapy alone, respectively. Of the 3 patients with adjuvant CCRT, 1 patient received 5-fluorouracil (5-FU) as the chemoregimen. The regimen performed in the remaining 2 cases could not be determined due to missing medical records. The regimen of 3 patients with curative intent resection followed by adjuvant chemotherapy alone was etoposide and cisplatin treatment.

**Figure 2:**
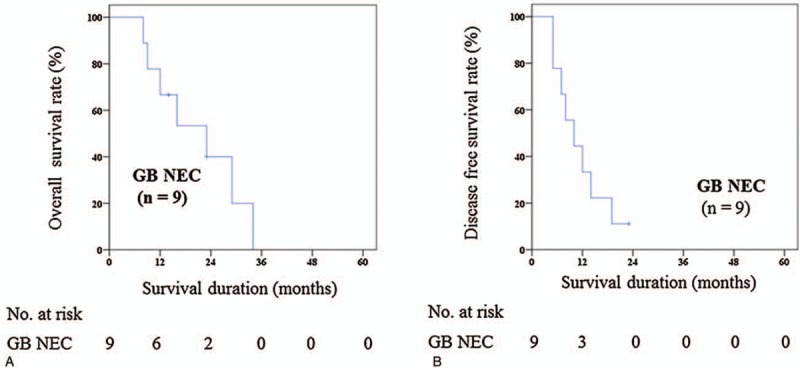
(A) 5ys overall survival rate (Tx.4—curative intent resection). (B) 5ys disease free survival rate (Tx.4—curative intent resection) . GB-NEC = gallbladder neuroendocrine carcinoma.

There were no in-hospital deaths and 90 days mortality among the 9 patients. Two patients were still alive during 14-month and 23-month follow-up period, respectively. Median overall survival and disease-free survival were 23.0 (8.0–34.0) and 10.0 (5.0–23.0) months, respectively (Fig. 2). The recurrence rate after curative-intent resection was 88.9%. The locoregional recurrence developed at the aortocaval space in 1 case. There were 8 identified sites of distant recurrence; 5 cases of intrahepatic area, 2 cases of distant lymph node, and 1 case of peritoneum, respectively. Among the 8 patients with recurrence, 3 patients were treated with palliative treatment. 2 patients were treated with etoposide and cisplatin. One patient was treated with etoposide and cisplatin-based CCRT.

### 3.3 Comparative analysis of treatment modality in GB-NEC

**Figure 3:**
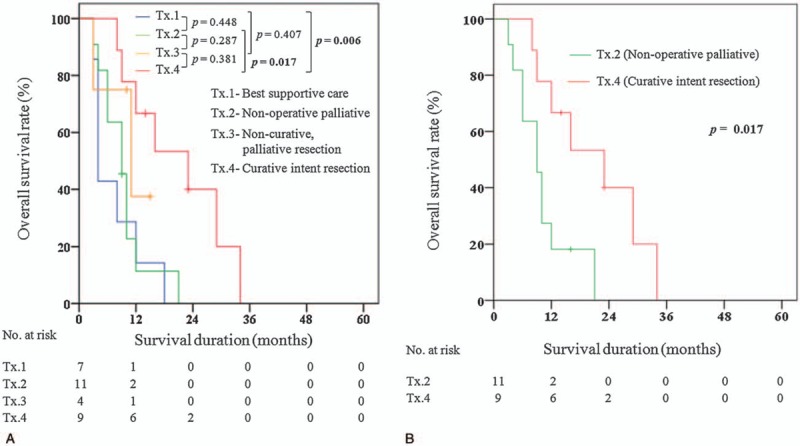
(A) 5 years overall survival rate—GB-NEC (all treatment). (B) 5 years overall survival rate—GB-NEC (Tx.2—non-operative palliative treatment versus Tx.4—curative intent resection).

Survival analysis according to treatment methods in 31 patients who were diagnosed with GB-NEC was shown in Figure 3A. The median overall survival between 4 groups was as follows: 4.0 (3.0–18.0) months in Tx.1 (n = 7) versus 9.0 (3.0–21.0) months in Tx.2 (n = 11) versus 11.0 (3.0–15.0) months in Tx.3 (n = 4) versus 23.0 (8.0–34.0) months in Tx.4 (n = 9), respectively. The median survival time of Tx.4 was significantly longer than Tx.1 and Tx.2, respectively (23.0 months vs 4.0 months, *P* = .006; 23.0 months vs 9.0 months, *P* = .017). Meanwhile, Tx.4 did not show better survival outcomes than Tx.3, significantly (23.0 months vs 11.0 months, *P* = .381). There was no significant difference in Tx.1 versus Tx.2, Tx.2 versus Tx.3, and Tx.1 versus Tx.3, respectively (*P* = .448, *P* = .287, and *P* = .407).

Clinicopathologic details of 11 cases of Tx.2 were compared to 9 cases of Tx.4, and with the exception of follow-up duration [9.6 (3.0–21.0) months versus18.6 (8.0–34.0) months, *P* = .021], a significant difference between 2 groups was not observed. Although the *P* value is more than .05, Tx.2 had a greater proportion of T3/T4 stage and positive regional lymph nodes metastasis than Tx.4. The difference of overall survival between 2 groups was statistically significant (*P* = .017) (Fig. 3B). The median overall survival was 9.0 (3.0–21.0) months in Tx.2and 23.0 (8.0–34.0) months in Tx.4, respectively.

## 4 Discussion

Several studies have reported dismal prognosis of GB-NEC with a median overall survival of 3 to 14 months.^[4,7,13–19]^ Most patients died within 3 years after diagnosis.^[4,14,15,18]^ Rare, but poor prognosis of GB-NEC was also shown in the present study. Many researchers suggested that poor oncologic outcome could be caused by aggressive biologic behavior of tumor and advanced stage at presentation. Chen et al's study represented that GB-NEC has a tendency of advanced disease progression at diagnosis, poor differentiation of tumor cells and lymphatic metastases compared to GBC.^[15]^ In the study, 9 of 10 cases in GB-NEC were stage IV and lymph node metastasis occurred in 70.0%. Kim et al reported cancer stage of all 7 patients diagnosed with GB-NEC after curative resection was IIIB or worse and more than half of total GB-NEC had metastasis of lymph nodes and liver.^[4]^ In our study, no patients were diagnosed with GB-NEC by the screening test. Insidious progression of disease and late diagnosis may contribute partly to the advanced stage of GB-NEC at detection. In addition, poor cell differentiation in all patients undergoing curative resection seems to have affected poor prognosis.

There has been an ongoing debate over whether the treatment of GB-NEC requires aggressive radical surgery in recent years. Some studies have reported skeptical results of surgical treatment. Lee et al showed indirect results suggesting that surgical treatment was not superior to chemotherapy.^[16]^ In the study, no significant difference in overall median survival after treatment for 6 GB-NEC versus 6 cases of MANEC was found (9.3 months vs 8.0 months, respectively; *P* = .997); 4 out of 6 in GB-NEC receiving chemotherapy and all 6 in MANEC undergoing surgical treatment had similar poor survival outcomes. Through literature review, Fujii et al suggested chemotherapy is effective and a useful modality of treatment to improve survival rather than surgery.^[14]^ A total of 53 patients in the study could not receive radical operations because of the characteristics of disease being diagnosed late and progressing to a severe stage. Inoperable states at diagnosis were also found in Kamboi et al's study.^[18]^ All 19 patients had distant metastasis at detection and were treated by palliative chemotherapy.

By contrast, other studies insisted that a surgical approach is one of the most important treatments for GB-NEC.^[15,17,20]^ Despite no established, widely accepted surgical strategy, many authors suggested that aggressive, radical cholecystectomy would be a method to improve survival.^[17,18,21]^ Kim et al reported 7 cases of GB-NEC with surgical resection.^[4]^ In the study, all patients underwent aggressive radical cholecystectomy; 3 received radical cholecystectomy and 4 extended cholecystectomy with liver lobectomy. However, there was no study that compared directly the curative intent resection to non-operative palliative treatment. The present study demonstrated that even if the chances of receiving curative intent resection are extremely low, the curative intent surgical approach has a significantly better outcome compared to non-operative palliative treatment. By contrast, palliative resection has no significant effect on improving median survival compared to the best supportive care and non-operative palliative treatment, respectively. Thus, for resectable cases, in which no distant metastasis on preoperative image work up or pathologic confirmation was found, curative-intent resection such as radical cholecystectomy should be given priority first as a treatment of GB-NEC. Considering the fact that late diagnosis takes away chances of getting curative intent resection, early detection of the disease should also be made, along with the efforts to accomplish curative resection.

In our study, despite significantly better outcomes of curative intent resection compared to non-operative palliative treatment, all patients expired within 3 years. In this regard, in order to improve survival rate after curative resection, development of effective and safe adjuvant treatment is urgently needed. In fact, there was no standardized protocol of adjuvant treatment for GB-NEC after curative surgery until now. The extremely low proportion of curative resection in previous studies made it difficult to verify the role of adjuvant treatment for the disease. Of course, there are no studies comparing directly the difference in oncologic outcomes after chemotherapy alone to CCRT. Although there was an opinion that NETs are insensitive to traditional radiotherapy,^[22]^ the effect of radiation after surgery in GB-NEC has not been disclosed due to paucity of data. Iype et al suggested cisplatin, gemcitabine, and etoposide plus 5-FU as the regimen of choice based on their study and previous literature review.^[23]^ In the present study, adjuvant treatment was performed in all cases with curative intent resection except for 1. They received a chemoregimen of etoposide and cisplatin or 5-FU and exhibited a median survival of 23.0 months. Given the small numbers of cases and the discordance of regimen of chemotherapy in the studies that have been conducted so far, the effects of adjuvant therapy on improving survival is still inconclusive. Therefore, the collaborative prospective studies enabling the analysis of a large number of data need to be conducted in order to prove the effect of adjuvant treatment and establish proper protocol, including type of agents, treatment duration and dose of drugs.

Concerning palliative treatment, previous studies reported marginal effect from an oncologic perspective. Elahi et al reported 46 months of survival time in a patient with postoperative chemotherapy.^[24]^ The chemotherapy regimens were gemcitabine plus cisplatin. After recurrence at the liver, treatment with docetaxel plus sunitinib and radiofrequency ablation was performed. However, extremely poor outcomes after palliative treatment were reported by a recent study.^[18]^ In the study, 12 of 19 patients who had distant metastasis could receive palliative chemotherapy but showed 3 months of medial survival. In the present study, median survival of the non-operative palliative treatment group could not demonstrate significantly better than those of best supportive care group (9.0 months vs 4.0 months, *P* = .448). Nonetheless, considering the rare possibility of curative resection attributable to the characteristics of the GB-NEC, research for identifying a proper regimen of palliative treatment that can delay the progression of disease should not be discontinued.

There are some limitations in this study. First, retrospective study design made it difficult to correct selection bias. Second, clinical outcome of patients might have been influenced by the absence of standardized adjuvant treatment protocol. Third, we could not performed multivariate analysis for risk factors due to small sample size. The design of a research study using a large sample size, such as a nationwide investigation, may be needed in the future.

## 5 Conclusions

Most of GB-NEC shows aggressive disease progression, a high rate of recurrence and poor survival. Nonetheless, curative-intent resection could possibly promote longer survival than other treatment modalities for GB-NEC. Given this, efforts to obtain chances of curative resection by early detection and further study about development of adjuvant treatment enabling the improvement in survival are needed.

## Author contributions

**Conceptualization:** Yung Hun You, Dong Wook Choi, Kee-Taek Jang.

**Data curation:** Yung Hun You, Dong Wook Choi, Seong Ho Choi, Jin Seok Heo, In Woong Han, Kee-Taek Jang, Sunjong Han.

**Formal analysis:** Yung Hun You, Dong Wook Choi, Seong Ho Choi, Jin Seok Heo, In Woong Han, Kee-Taek Jang, Sunjong Han.

**Investigation:** Yung Hun You.

**Methodology:** Yung Hun You, Dong Wook Choi.

**Resources:** Yung Hun You.

**Supervision:** Yung Hun You, Dong Wook Choi, Seong Ho Choi, Jin Seok Heo, In Woong Han, Kee-Taek Jang.

**Writing – original draft:** Yung Hun You.

**Writing – review & editing:** Dong Wook Choi, Seong Ho Choi, Jin Seok Heo, In Woong Han, Sunjong Han.

## References

[1] Modlin IM Lye KD Kidd M A 5-decade analysis of 13,715 carcinoid tumors. Cancer 2003;97:934–59. 12569593 10.1002/cncr.11105

[2] Rothenstein J Cleary SP Pond GR Neuroendocrine tumors of the gastrointestinal tract: a decade of experience at the Princess Margaret Hospital. Am J Clin Oncol 2008;31:64–70. 18376230 10.1097/COC.0b013e31807a2f49

[3] Chamberlain RS Blumgart LH Carcinoid tumors of the extrahepatic bile duct. A rare cause of malignant biliary obstruction. Cancer 1999;86:1959–65. 10570419

[4] Kim J Lee WJ Lee SH Clinical features of 20 patients with curatively resected biliary neuroendocrine tumours. Dig Liver Dis 2011;43:965–70. 21856258 10.1016/j.dld.2011.07.010

[5] Gustafsson BI Kidd M Modlin IM Neuroendocrine tumors of the diffuse neuroendocrine system. Curr Opin Oncol 2008;20:1–2. 18043250 10.1097/CCO.0b013e3282f1c595

[6] Yao JC Hassan M Phan A One hundred years after “carcinoid": epidemiology of and prognostic factors for neuroendocrine tumors in 35,825 cases in the United States. J Clin Oncol 2008;26:3063–72. 18565894 10.1200/JCO.2007.15.4377

[7] Eltawil KM Gustafsson BI Kidd M Neuroendocrine tumors of the gallbladder: an evaluation and reassessment of management strategy. J Clin Gastroenterol 2010;44:687–95. 20375728 10.1097/MCG.0b013e3181d7a6d4

[8] Bosman FT Carneiro F Hruban RH WHO Classification of Tumours of the Digestive System. 4th ed. 2010;Lyon: The International Agency for Research on Cancer, 3:13–14, in press.

[9] Modlin IM Shapiro MD Kidd M An analysis of rare carcinoid tumors: clarifying these clinical conundrums. World J Surg 2005;29:92–101. 15599742 10.1007/s00268-004-7443-z

[10] Jun SR Lee JM Han JK High-grade neuroendocrine carcinomas of the gallbladder and bile duct: report of four cases with pathological correlation. J Comput Assist Tomogr 2006;30:604–9. 16845291 10.1097/00004728-200607000-00009

[11] Lane JE Walker AN Ayers GW Small-cell undifferentiated carcinoma of neuroendocrine type originating in the gallbladder. Curr Surg 2002;59:495–7. 15727797 10.1016/s0149-7944(02)00638-4

[12] Beasley MB Brambilla E Travis WD The 2004 World Health Organization classification of lung tumors. Semin Roentgenol 2005;40:90–7. 15898407 10.1053/j.ro.2005.01.001

[13] Duffy A Capanu M Abou-Alfa GK Gallbladder cancer (GBC): 10-year experience at Memorial Sloan-Kettering Cancer Centre (MSKCC). J Surg Oncol 2008;98:485–9. 18802958 10.1002/jso.21141

[14] Fujii H Aotake T Horiuchi T Small cell carcinoma of the gallbladder: a case report and review of 53 cases in the literature. Hepatogastroenterology 2001;48:1588–93. 11813580

[15] Chen C Wang L Liu X Gallbladder neuroendocrine carcinoma: report of 10 cases and comparision of clinicopathologic features with gallbladder adenocarcinoma. Int J Clin Exp Pathol 2015;8:8218–26. 26339390 PMC4555718

[16] Lee JM Hwang S Lee SG Neuroendocrine tumors of the gallbladder: twelve cases in a single institution. Hepatogastroenterology 2010;57:1064–8. 21410032

[17] Yun SP Shin N Seo HI Clinical outcomes of small cell neuroendocrine carcinoma and adenocarcinoma of the gallbladder. World J Gastroenterol 2015;21:269–75. 25574101 10.3748/wjg.v21.i1.269 PMC4284345

[18] Kamboj M Gandhi JS Gupta G Neuroendocrine carcinoma of gall bladder: a series of 19 cases with review of literature. J Gastrointest Cancer 2015;46:356–64. 26208508 10.1007/s12029-015-9745-9

[19] Moskal TL Zhang PJ Nava HR Small cell carcinoma of the gallbladder. J Surg Oncol 1999;70:54–9. 9989422 10.1002/(sici)1096-9098(199901)70:1<54::aid-jso10>3.0.co;2-w

[20] Modlin IM Kidd M Latich I Current status of gastrointestinal carcinoids. Gastroenterology 2005;128:1717–51. 15887161 10.1053/j.gastro.2005.03.038

[21] Reid KM Ramos-De la Medina A Donohue JH Diagnosis and surgical management of gallbladder cancer: a review. J Gastrointest Surg 2007;11:671–81. 17468929 10.1007/s11605-006-0075-x

[22] Modlin IM Kidd M Drozdov I Pharmacotherapy of neuroendocrine cancers. Expert Opin Pharmacother 2008;9:2617–26. 18803449 10.1517/14656566.9.15.2617

[23] Iype S Mirza TA Propper DJ Neuroendocrine tumours of the gallbladder: three cases and a review of the literature. Postgrad Med J 2009;85:213–8. 19417172 10.1136/pgmj.2008.070649

[24] Elahi F Ahmadzadeh A Yadollahzadeh M Neuroendocrine tumor of the gallbladder. Arch Iran Med 2013;16:123–5. 23360636

